# The efficacy and safety of pre-emptive methoxamine infusion in preventing hypotension by in elderly patients receiving spinal anesthesia: A PRISMA-compliant protocol for systematic review and meta-analysis

**DOI:** 10.1097/MD.0000000000032262

**Published:** 2022-12-09

**Authors:** Ling Li, Li-Xian He, Yun-Tai Yao

**Affiliations:** a Department of Anesthesiology, Fuwai Yunnan Cardiovascular Hospital, Kunming, Yunnan Province, China; b Anesthesia Center, Fuwai Hospital, NCCD, PUMC&CAMS, Beijing, China.

**Keywords:** elderly patients, hypotension, meta-analysis, methoxamine, spinal anesthesia

## Abstract

Hypotension is frequent after spinal anesthesia, especially in elderly patients. Whether pre-emptive methoxamine infusion is effective and safe to prevent spinal anesthesia-induced hypotension is still a controversial issue, to dress this knowledge lack, we performed a systemic review and meta-analysis to evaluated it.

**Participants:**

Elderly patients undergoing spinal anesthesia.

**Interventions:**

Administration of methoxamine prior to spinal anesthesia.

**Methods:**

We searched PUBMED, Cochrane Library, EMBASE, China National Knowledge Infrastructure, Wanfang Database, and VIP Database, Chinese BioMedical Literature & Retrieval System from January 1^st^ 1978 to February 28^th^ 2022. Primary outcomes of interests included hemodynamic parameters, such as systolic blood pressure, diastolic blood pressure, mean arterial pressure, heart rate. Secondary outcomes of interests included the incidence of intraoperative hypotension, bradycardia, nausea and vomiting, vasopressors requirement, intraoperative blood loss. For continuous or dichotomous variables, treatment effects were calculated as weighted mean difference or odds ratio, respectively.

**Results:**

Our search yielded 8 randomized controlled trials including 480 patients, and 240 patients were allocated into methoxamine group and 240 into control group. Meta-analysis demonstrated that pre-emptive methoxamine infusion in preventing hypotension by in elderly patients receiving spinal anesthesia had higher blood pressures, lower heart rates. Compared with the control group, the incidence of perioperative hypotension in elderly patients was lower, and elderly patients had less requirement for vasopressor in methoxamine group.

**Conclusion:**

This meta-analysis demonstrated that pre-emptive methoxamine infusion in elderly patients receiving spinal anesthesia can improve blood pressure, slow down heart rate, reduce the incidence of hypotension and requirement for vasopressor. However, these findings should be interpreted rigorously. Further well-conducted trials are required to confirm this.

## 1. Introduction

Spinal anesthesia (SA) induces sympathetic nerve blockade and causes vasodilation, leads hypotension.^[1]^ The reported incidence of hypotension after SA in elderly patients was as high as 73% due to their decreased functional reserve and poor vascular elasticity.^[2,3]^ Elderly patients who are usually accompanied by cardiovascular diseases, are intolerant to hypotension. If spinal anesthesia-induced hypotension (SAIH) cannot be effectively prevented, it might lead to serious adverse events, and even increase the perioperative mortality in elderly patients.^[4–6]^

Vasopressors are often used to prevent SAIH. However, administration of commonly-used vasopressors such as ephedrine, dopamine, norepinephrine, etc, enhance blood pressure and increase both heart rate as well.^[7,8]^ Unlike them, methoxamine (MX) is highly selective α_1_ receptor agonist, which mainly acts on α_1_A and α_1_B receptors distributed in peripheral blood vessels, but have little effect on α_1_D receptors distributed in coronary arteries.^[9,10]^ MX can enhance blood pressure while reflexively slowing down heart rate, increase coronary blood flow, improve myocardial oxygen supply, and reduce myocardial oxygen consumption, which is beneficial to the elderly. MX has drawn attention due to its unique receptor effect and it has been widely used in China to prevent and treat various perioperative hypotension in surgical patients. Evidence has accumulated that pre-emptive MX infusion can effectively maintain the stability of perioperative hemodynamics in elderly patients undergoing hip replacement, femoral head replacement, gynecological surgery, radical gastrectomy, prostate resection, and coronary artery bypass graft surgery.^[11–18]^ Furthermore, studies have demonstrated that MX is effective in preventing hypotension after induction of general anesthesia.^[19–24]^ However, there are also reports of severe bradycardia caused by MX.^[25,26]^ As the evidence of the safety and efficacy of MX in preventing hypotension after SA in elderly patients remains undetermined. Therefore, we performed this meta-analysis to evaluate the efficacy and safety of pre-emptive MX infusion in preventing hypotension by in elderly patients receiving SA.

## 2. Methods

### 2.1. Ethical approval

This study was a meta-analysis of previously published literature; ethical approval was not necessary according to the Ethical Committee of Fuwai Hospital.

### 2.2. Search strategy

We conducted a systemic review according to the Preferred Reporting Items for Systemic Reviews and Meta-Analysis Quality of Reporting of Meta-analysis (PRIMSA) Guidelines.^[27]^ The protocol of current meta-analysis was published in PROSPERO with the registration number of CRD42020163270. Relevant trials were identified by computerized searches of PUBMED, Cochrane Library, EMBASE, China National Knowledge Infrastructure, VIP Database, and Wangfang Database from January 1^st^ 1978 to February 28^th^ 2022, using different combination of search words as follows: *(Methoxamine odds ratio [OR] Vasxine OR Vasoxyl) AND (spinal anesthesia OR subarachnoid anesthesia) AND (randomized controlled trial OR controlled clinical trial OR randomized OR placebo OR randomly OR trial*) (Appendix). No language restriction was used. We also searched() Chinese Bio Medical Literature & Retrieval System (from January 1^st^ 1978 to February 28^th^ 2022). Additionally, we used the bibliography of retrieved articles to further identify relevant studies.

### 2.3. Inclusion and exclusion criteria

We included all clinical trials comparing the MX with controls (vasopressors/blank) on elderly patients underwent SA. Primary outcomes of interest included systolic blood pressure (SBP), diastolic blood pressure (DBP), mean arterial pressure (MAP), heart rate (HR). Secondary outcomes of interest include the incidence of intraoperative hypotension, bradycardia, nausea and vomiting, requirement for vasopressor, intraoperative blood loss, etc. Exclusion criteria included studies published as review, case report or abstract; animal or cell studies; duplicate publications; studies lacking information about outcomes of interest. Two authors (LL and LXH) independently reviewed the titles and abstracts of all identified studies for eligibility, excluding obviously ineligible ones. The eligibility of those remaining studies for final inclusion was further determined by reading the full text.

### 2.4. Study quality assessment

Two authors (LL and YTY) will independently assess the risk of bias, using the tool described in the Cochrane Handbook for Systematic Reviews of interventions.^[28]^ The modified *Jadad* score^[29]^ will also be used independently by 2 authors (LL and YTY) to evaluate the methodological quality of each included trial.

### 2.5. Data abstraction

The following data were abstracted from the included studies to a data collection form by 2 authors (YTY and LXH) independently: author, year of publication and journal of included studies; total number of patients, number of patients in MX and control (CTL) groups, gender, age and body weight; local anesthetics used for SA; data regarding outcomes of interest in both groups. Disagreements were resolved by discussion among all authors during the process of data abstraction.

### 2.6. Statistical analysis

All data were analyzed by utilizing RevMan 5.4 (Cochrane Collaboration, Oxford, UK). Pooled odds ratio (OR) and 95% confidence interval (CI) were estimated for dichotomous data, and weighted mean difference (WMD) and 95% CI for continuous data, respectively. Each outcome was tested for heterogeneity, and randomized-effects or fixed-effects model was used in the presence or absence of significant heterogeneity (*Q*-statistical test *P* < .05). Sensitivity analyses were done by examining the influence of statistical model on estimated treatment effects, and analyses which adopted the fixed-effects model were repeated again by using randomized-effects model and vice versa. In addition to that, sensitivity analysis was also performed to evaluate the influence of individual study on the overall effects. The possible effects of administration methods will be evaluated by subgroup analysis. Publication bias was explored through visual inspection of funnel plots of the outcomes. All *P* values were 2-sided and statistical significance was defined as *P* < .05.

## 3. Results

### 3.1. Search results

**Figure 1.**
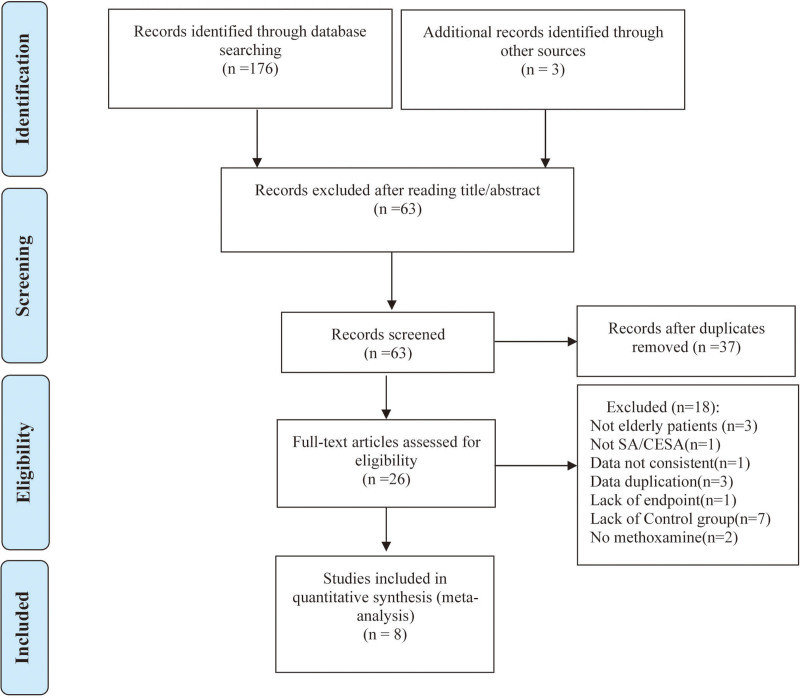
Flow chart.

As depicted in the flow chart (Fig. 1), a database search identified 26 articles^[15,17,30–53]^ for complete evaluation. Finally, 8 eligible trails^[15,30,33–35,37,49,50]^ including 480 patients were enrolled in the meta-analysis. Among them, 240 patients were allocated into the MX group and 240 into the CTL group. Five trials^[30,33,35,49,50]^ were blank CTL, 3 trials^[15,34,37]^ included ephedrine. Of the 8 trials, 1^[30]^ were performed in Ireland (written in English), 7^[15,33-35,37,49,50]^ in China (written in Chinese). Descriptive analyses of these articles are shown Table 1.

### 3.2. Included trial characteristics

AS shown Table 1, 6 studies^[33–35,37,49,50]^ included patients with lower limb orthopedic surgery procedures, 1 studies^[30]^ included patients who undergoing transurethral resection of the prostate (TURP), and another study^[15]^ included hysteromyomectomy patients. Patients in 2 trials^[30,33]^ were administered MX intramuscularly and 6^[15,34,35,37,49,50]^ were administered MX intravenously. Patient characteristics were presented in Table S1, Supplemental Digital Content, http://links.lww.com/MD/I104. All of them were elderly patients, with the maximum age of 92 and the minimum age of 59, and ASA grade I–III.

**Table 1:** Characteristics of included trials.

Trials	Country	Surgery	MX group		Control group			Outcomes
			Dosages	n	Control	Dosages	n	
Chambers1994^[30]^	Ireland	TURP	10 mg	17	BLK	–	19	a,b
He 2012^[33]^	China	Hip replacement	0.1mg/kg	30	BLK	–	30	a
Lin 2012^[34]^	China	Hip replacement	1 mg	20	Ephedrine	3 mg	20	a
Chen 2012^[35]^	China	ORIF	3 mg	40	BLK	–	40	a,b
Shang 2014^[37]^	China	ORIF OR FHR	2 mg	34	Ephedrine	5 mg	33	a,b,c,d
Fu 2018^[15]^	China	Hysteromyomectomy	1 mg	30	Ephedrine	5 mg	30	a
Jing 2019^[49]^	China	FHR	2 (+2.0 to 3.0 μg/kg/min)	29	BLK	–	28	a
Wang 2019(a)^[50]^	China	PFNA	2 mg	20	BLK	–	20	a,b
Wang 2019(b)^[50]^	China	PFNA	1 (+0.5 μg/kg/min)	20	BLK	–	20	a,b

BLK = blank, FHR = femoral head replacement, MX = methoxamine, ORIF = open reduction and internal fixation, PFNA = proximal femoral nail antirotation, TURP = transurethral resection of the prostate.

a Hemodynamics after SA.

b Incidence of hypotension.

c Incidence of bradycardia.

d Other complications.

### 3.3. Study quality and risk bias

The risk of bias analysis was shown in Figure S1, Supplemental Digital Content, http://links.lww.com/MD/I110 and Figure S2, Supplemental Digital Content http://links.lww.com/MD/I111 Randomization was used in 7 trials,^[15,30,33,35,37,50]^ 1 study^[49]^ did not mention it. All trials didn’t mention the blindness method and were at unclear risk of bias. The modified *Jadad* scores of the 8 included RCTs ranged from 4-to-5, with all RCTs scored as “high quality” (Table S2, Supplemental Digital Content, http://links.lww.com/MD/I105).

### 3.4. Hemodynamics after SA

As shown in Table 2, meta-analysis presented the hemodynamic parameters were comparable after anesthesia between MX group and CTL group. Five minutes after SA, patients in MX group had higher DBP (weighted mean difference [WMD] = 6.97, 95% confidence interval [95% CI]: 1.22–12.73; *P* = .02), higher MAP (WMD = 8.37, 95% CI: 3.05–13.69; *P* < .00001), lower HR (WMD = –6.71, 95% CI: –11.65 to –1.76; *P* = .008). Ten minutes after SA, patients in MX group had higher SBP (WMD = 13.31, 95% CI: 6.53–20.09; *P* = .0001), higher DBP (WMD = 11.82, 95% CI: 8.29–15.35; *P* < .00001), higher MAP (WMD = 10.52, 95% CI: 7.39–13.64; *P* < .00001), lower HR (WMD = –12.87, 95% CI: –21.41 to –4.33; *P* = .003). Fifteen minutes after anesthesia, patients in MX group had higher SBP (WMD = 12.15, 95% CI: 3.01–21.30; *P* = .009), higher DBP (WMD = 13.55, 95% CI: 12.30–14.79; *P* < .00001), lower HR (WMD = –9.13, 95% CI: –14.19 to –4.06; *P* = .006). Fifteen minutes after anesthesia, patients in MX group had higher SBP (WMD = 11.25, 95% CI: 4.33–18.18; *P* = .001), higher DBP (WMD = 9.67, 95% CI: 5.28–14.05; *P < *.0001), lower HR (WMD = –8.55, 95% CI: –13.02 to –4.09; *P* = .0002). Twenty minutes after anesthesia, patients in MX group had higher SBP (WMD = 10.00, 95% CI: 2.15–17.85; *P* = .01), higher MAP (WMD = 9.01, 95% CI: 6.57–11.45; *P* < .00001). Thirty minutes after anesthesia, patients in MX group had higher SBP (WMD = 10.39, 95% CI: 6.31–14.47; *P* < .00001), higher DBP (WMD = 12.90, 95% CI: 3.58–22.21; *P* = .007), higher MAP (WMD = 8.31, 95% CI: 6.04–10.58; *P* < .00001), lower HR (WMD = -8.53, 95% CI: -14.75– -2.31; *P* = .007).

**Table 2:** Meta-analysis of hemodynamics after SA.

	Trails/Comparisons (n)	Total (n)	MX (n)	CTL (n)	WMD	95% CI	*I* ^2^	Heterogeneity *P*	Model	Overall effect *P*
**SBP**										
Baseline	6/6	364	183	181	–1.22	–5.02, 2.58	0%	.98	FEM	.53
5 min after SA	5/5	307	154	153	8.66	–0.84, 18.17	89%	<.00001	REM	.07
10 min after SA	3/3	200	100	100	13.31	6.53, 20.09	0%	.04	FEM	.0001
15 min after SA	6/6	364	183	181	12.15	3.01, 21.30	86%	<.00001	REM	.009
20 min after SA	1/1	60	30	30	10.00	2.15, 17.85	NA	NA	NA	.01
30 min after SA	2/2	127	64	63	10.39	6.31, 14.47	0%	.32	FEM	<.00001
**DBP**										
Baseline	5/5	307	154	153	–1.96	–4.44, 0.52	0%	.95	FEM	.12
5 min after SA	5/5	307	154	153	6.97	1.22, 12.73	81%	.0003	REM	.02
10 min after SA	3/3	200	100	100	11.82	8.29, 15.35	57%	.10	REM	<.00001
15 min after SA	5/5	307	154	153	13.55	12.30, 14.79	85%	<.0001	REM	<.00001
20 min after SA	1/1	60	30	30	10.00	4.91, 15.09	NA	NA	NA	.0001
30 min after SA	2/2	127	64	63	12.90	3.58, 22.21	89%	.002	REM	.007
**MAP**										
Baseline	2/3	120	60	60	–1.54	–3.80, 0.72	0%	.72	FEM	.18
5 min after SA	2/3	120	60	60	8.37	3.05, 13.69	70%	.002	FEM	<.00001
10 min after SA	1/2	80	40	40	10.52	7.39, 13.64	0%	.72	FEM	<.00001
15 min after SA	2/2	78	37	39	2.72	–4.01, 9.45	53%	.15	REM	.43
20 min after SA	1/2	80	40	40	9.01	6.57, 11.45	0%	.40	FEM	<.00001
30 min after SA	1/2	80	40	40	8.31	6.04, 10.58	26%	.25	FEM	<.00001
**HR**										
Baseline	7/8	444	223	221	–0.69	–2.18, 0.79	10%	.35	FEM	.36
5 min after SA	6/7	387	194	193	–6.71	–11.65, –1.76	89%	<.00001	REM	.008
10 min after SA	4/5	280	140	140	–12.87	–21.41, –4.33	94%	<.00001	REM	.003
15 min after SA	7/7	393	196	197	–8.55	–13.02, –4.09	91%	<.00001	REM	.0002
20 min after SA	2/3	140	70	70	–4.58	–11.72, 2.56	85%	.002	REM	.21
30 min after SA	3/4	207	104	103	–8.53	–14.75, –2.31	87%	<.0001	REM	.007

CTL = control, DBP = diastolic blood pressure, FEM = fixed effect model, HR = heart rate, MAP = mean arterial pressure, NA = not applicable, WMD = weighted mean difference, OR = odds ratio, REM = random effect model, SBP = systolic blood pressure, SA = spinal anesthesia, 95% CI = 95% confidence interval.

### 3.5. Subgroups analysis

Meta-analysis suggested that MX had similar hemodynamics effects on these 2 subgroups patients after anesthesia except for outcomes shown in Supplemental Table S3, Supplemental Digital Content, http://links.lww.com/MD/I106. And subgroup analysis of different administration methods generated similar results except for outcomes shown in supplemental Table S4, Supplemental Digital Content, http://links.lww.com/MD/I107.

### 3.6. Hypotension after SA

**Figure 2.**
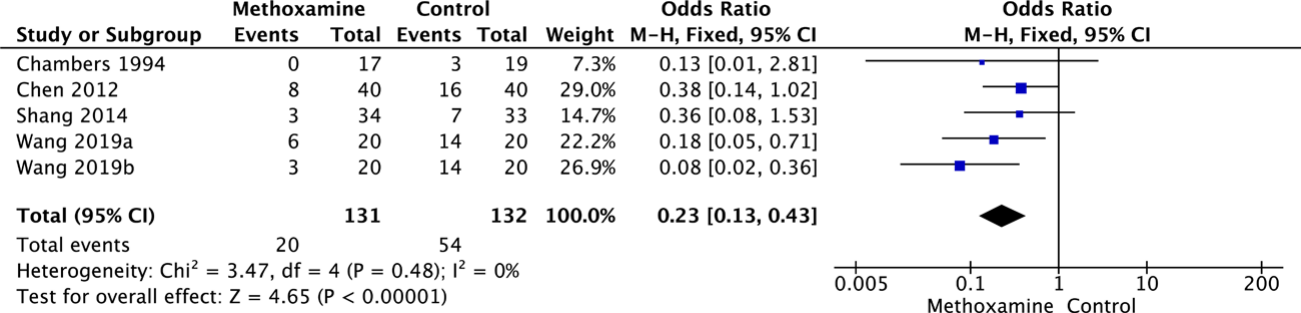
Forest plot of hypotension.

As shown in Figure 2, 4 trials,^[30,35,37,50]^ (263 patients) reported the incidence of hypotension after anesthesia, and meta-analysis suggested that the incidence of hypotension in MX group was lower than that in CTL group (15.3% vs 40.10%, OR = 0.23, 95% CI: 0.13–0.43; *P* < .00001). One trails^[37]^ (67 patients) reported the occurrence of nausea and vomiting (23.5% vs 33.3%; OR = 0.62; 95% CI: 0.21–1.80; *P* = .38) and the incidence of bradycardia (26.5% vs 12.1%; OR = 2.61; 95%CI: 0.72–9.52; *P* = .15) after anesthesia, meta-analysis suggested that were similar in MX group patients and CTL group patients.

### 3.7. Requirement for vasopressor

**Figure 3.**
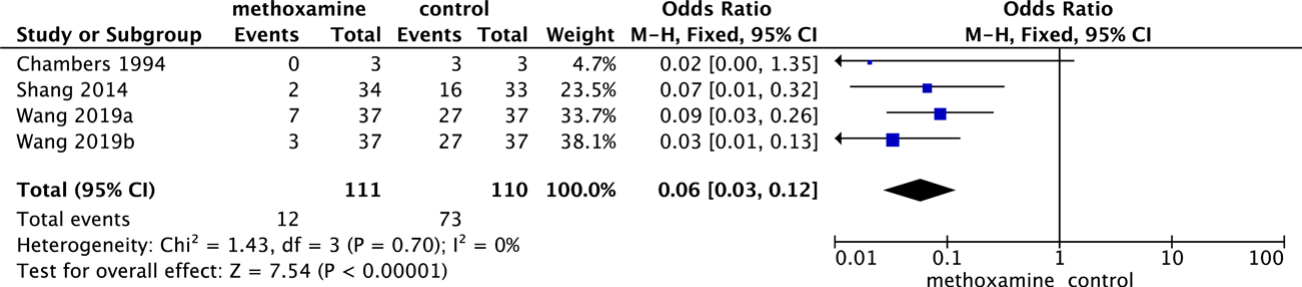
Forest plot of vasopressor requirement.eps.

As shown in Figure 3, 4 trials^[30,37,50]^ (221 patients) suggested times of adding vasopressor in a single time, meta-analysis demonstrated that patients with pre-emptive MX infusion had less requirement for a vasopressor (10.81% vs 66.36%; OR = 0.06; 95%CI: 0.03–0.12; *P* < .00001) after anesthesia compared with patients in CTL group.

### 3.8. Sensitivity analyses and publication

Sensitivity analysis showed that the treatment effects were not affected by the choice of statistical model, except for the outcomes shown in Table S5, Supplemental Digital Content, http://links.lww.com/MD/I108. In addition, sensitivity tests also were performed by exclusion of some studies to analyze the influence of the overall treatment effect on high heterogeneity outcomes. And there are contradictory results in outcomes shown in supplemental Table S6, Supplemental Digital Content, http://links.lww.com/MD/I109. No significant publication bias was detected by funnel plot examination for the incidence of hypotension (Figure S3, Supplemental Digital Content, http://links.lww.com/MD/I112).

## 4. Discussion

SAIH is frequent due to arterial and venous vasodilatation resulting from the sympathetic block along with a paradoxical activation of cardioinhibitory receptors, which usually has potential adverse consequences. Elderly patients have decreased physiological organ function reserve, impaired sympathetic nerve regulation, decreased arterial elasticity, and the incidence of SAIH is higher. Furthermore, elderly patients usually complicated with coronary heart disease, cerebral infarction, vascular sclerosis and stenosis, hypotension can result in hypoperfusion of vital organs and a serious epiphenomenon will occur.^[2,53–55]^ Therefore, preventing SAIH and maintaining hemodynamic stability during the perioperative period is very important for elderly patients.

MX, α_1_ adrenoreceptor agonist, is a commonly used vasopressor in clinic. α_1_ receptors are divided into three subtypes: α_1_A, α_1_B, and α_1_D. MX predominantly acts on α_1_A and α_1_B receptors which has obvious contractive effect on peripheral blood vessels, but have little effect on α_1_D receptors distributed in coronary arteries. MX plays the following roles: increase peripheral vascular resistance, increase hypertension, reduce myocardial oxygen consumption, increase myocardial perfusion pressure and coronary blood flow, and increase myocardial oxygen supply.^[56]^ Therefore, compared with other vasopressors, MX is more suitable for elderly patients with coronary heart disease.^[18,57,58]^ At the same time, MX is recommended as the first-line drug in patients with outflow tract obstruction such as hypertrophic cardiomyopathy, valve stenosis, heart failure, etc.^[56]^

MX is widely used in the prevention and treatment of hypotension in perioperative elderly patients in China.^[11–24]^ To our knowledge, this is the first meta-analysis to evaluate the safety and efficacy of MX in preventing SAIH in elderly patients. The present meta-analysis suggested that, compared with the control group, the pre-emptive MX infusion in preventing hypotension by in elderly patients receiving SA had higher blood pressure, lower heart rate and lower incidence of hypotension, and the application of MX resulted in more stable hemodynamics in elderly patients. The current meta-analysis also showed that the elderly patients in the MX group had less demand for single dose of vasopressor. It is noteworthy that the worrisome side effects of MX, bradycardia, need further investigation. On the one hand, sympathetic block and vagal reflex can induce bradycardia after SA.^[59,60]^ On the other hand, reflective bradycardia caused by MX increasing blood pressure. Of the randomized controlled trials included in this meta-analysis, only 1^[37]^ mentioned, the incidence of bradycardia in MX group was similar with CTL group, there was no difference between the doses of atropine and no report of severe bradycardia. The studies^[18,57]^ of MX used to prevent hypotension during coronary artery bypass grafting demonstrated that MX pretreatment can reduce the hemodynamic fluctuation after induction of general anesthesia, the incidence of hypotension is lower than that of patients without MX pretreatment, there was no significant change in heart rate between the 2 groups during perioperative period and there was no significant difference in the use of agents to improve heart rate. The randomized controlled study conducted by Sun et al^[25]^ on the effect of MX on the hemodynamics of painless gastrointestinal endoscopy showed that the incidence of bradycardia in patients in the MX group increased immediately after induction administration, no adverse consequences after observation or intravenous injection of atropine. It is considered that it is related to the reflex slowing of carotid sinus baroreceptor and the excessive speed of intravenous MX injection. Therefore, pre-injection of MX slowly may benefit elderly patients more. The case report of Zhou et al^[26]^ showed that severe bradycardia may occur when patients were given large doses of MX. As a result, it is generally recommended to give a single dose of 1–2 mg intravenously before or at the same time of anesthesia. Continuous pumping during operation can also provide more lasting stable circulation, and the recommended dose is 1.5–4.0 µg/kg/minutes.^[56]^

Several limitations of the present meta-analysis should be considered. Meta-analysis can increase the power of analysis by pooling many small low-quality studies, but different administration modalities of MX (e.g., agent, dose, route, timing), varied quality and heterogeneity of the included studies, and possible biases may limit the certainty of the findings of meta-analysis. There were significant differences among the 8 clinical trials included in the meta- analysis with respect to sample size, study design, outcome definition, etc. In addition, these studies lack indicators such as intraoperative blood loss and long-term prognosis, so the findings should be interpreted rigorously. Further well-conducted trials are required to evaluate the efficacy and safety of preemptive MX infusion.

## 5. Conclusions

This meta-analysis has found some evidence showing that preemptive MX infusion in elderly patients receiving SA can improve blood pressure, slow down heart rate, reduce the incidence of hypotension and requirement for vasopressors. However, these findings should be interpreted rigorously. Further well-conducted trials are required to evaluate the efficacy and safety of preemptive MX infusion.

## Author contributions

**Conceptualization:** Yun-Tai Yao.

**Data curation:** Ling Li, Li-Xian He.

**Formal analysis:** Ling Li, Yun-Tai Yao.

**Investigation:** Ling Li, Yun-Tai Yao, Li-Xian He.

**Methodology:** Ling Li, Yun-Tai Yao, Li-Xian He.

**Project administration:** Yun-Tai Yao.

**Software:** Ling Li, Yun-Tai Yao, Li-Xian He.

**Supervision:** Yun-Tai Yao.

**Validation:** Yun-Tai Yao.

**Writing – original draft:** Ling Li, Li-Xian He.

**Writing – review and editing:** Yun-Tai Yao, Ling Li.

## Supplementary Material


