# Supplementary material for: The efficacy and safety of pre-emptive methoxamine infusion in preventing hypotension by in elderly patients receiving spinal anesthesia: A PRISMA-compliant protocol for systematic review and meta-analysis
Source: Medicine (Baltimore). 2022 Dec 9;101(49):e32262. doi: 10.1097/MD.0000000000032262 (PMC9750677; doi:10.1097/MD.0000000000032262)
Supplement: Supplementary file 1 [file medi-101-e32262-s001.pdf]

Supplemental Table 1. Characteristics of included patients

Trails	ASA	Age		Sex (male/female)		Body Weight (Kg)		Spinal Anesthesia		
		MX	CTL	MX	CTL	MX	CTL	Anesthetics	Dosages (ml/mg)	Injection Speed
Chambers1994[30]	NR	72.5(64-83)	72.4(59-82)	17/0	19/0	76.4±3.2	72.3±2.8	0.5% bupivacaine	2.5-3ml	NR
Chen 2012[35]	□~□	> 65		NR	NR	50-70	50-70	bupivacaine	10mg	0.08 ml/s
He 2012[33]	□~□	69-92	65-88	NR	NR	43-75	45-72	ropivacaine	9-12mg	NR
Lin 2012[34]	□~□	79.1±6.6	78.0±5.2	14/6	13/7	60.1±5.9	58.9±9.7	0.5% ropivacaine	7-12mg	NR
Shang 2014[37]	□~□	60-85		NR	NR	50-85		0.75% ropivacaine	8-12mg	20-30 s
Fu 2018[48]	□~□	61-82		0/30	0/30	48-77		0.75% bupivacaine	2ml(12-15mg)	at a constant rate
Jing 2019[51]	NR	69.8±3.34(61-76)	70.13±3.71(62-77)	18/11	16/12	NR	NR	ropivacaine	10-15mg	NR
Wang 2020(a)[52]	□~□	75.05±5.87	77.90±5.11	9/11	10/10	NR	NR	ropivacaine	2.5-3ml	Slow
Wang 2020(b)[52]	□~□	77.00±4.81	77.90±5.11	11/9	10/10	NR	NR	ropivacaine	2.5-3ml	Slow

Abbreviations: ASA = American Society of Anesthesiologists; CTL = control; MX = methoxamine; NR = not reported.
