# Supplementary material for: The efficacy and safety of pre-emptive methoxamine infusion in preventing hypotension by in elderly patients receiving spinal anesthesia: A PRISMA-compliant protocol for systematic review and meta-analysis
Source: Medicine (Baltimore). 2022 Dec 9;101(49):e32262. doi: 10.1097/MD.0000000000032262 (PMC9750677; doi:10.1097/MD.0000000000032262)
Supplement: Supplementary file 2 [file medi-101-e32262-s002.pdf]

Supplemental Table 2. Quality assessment of included studies

Studies	Modified *Jadad* Score				
	Randomization	Allocation	Blindness	Withdraws	Total
Jing 2019[51]	1	1	1	1	4
Wang 2019[52]	1	1	1	1	4
Fu 2018[48]	1	1	1	1	4
Shang 2014[37]	1	1	1	1	4
He 2012[33]	2	1	1	1	5
Chen 2012[35]	1	1	1	1	4
Lin 2012[34]	2	1	1	1	5
Chambers 1994[30]	2	1	1	1	5
