# Supplementary material for: The efficacy and safety of pre-emptive methoxamine infusion in preventing hypotension by in elderly patients receiving spinal anesthesia: A PRISMA-compliant protocol for systematic review and meta-analysis
Source: Medicine (Baltimore). 2022 Dec 9;101(49):e32262. doi: 10.1097/MD.0000000000032262 (PMC9750677; doi:10.1097/MD.0000000000032262)
Supplement: Supplementary file 3 [file medi-101-e32262-s003.pdf]

Supplemental Table 3. Subgroup analysis of hemodynamic indexes in blank control and ephedrine patients

Outcomes	Trails/Comparisons(n)	Total(n)	MX(n)	CTL(n)	WMD	95%CI	I^2	Heterogeneity *P*	Model	Overall Effect p
SBP										
Baseline										
MX *vs* BLK	3/3	197	99	98	-1.60	-6.74, 3.54	0%	0.97	FEM	0.54
MX *vs* Ephedrine	3/3	167	84	83	-0.77	-6.42, 4.89	0%	0.73	FEM	0.79
Total	6/6	364	183	181	-1.22	-5.02, 2.58	0%	0.98	FEM	0.53
5 min after SA										
MX *vs* BLK	2/2	140	70	70	10.29	6.35, 14.23	0%	0.82	FEM	<0.00001
MX *vs* Ephedrine	3/3	167	84	83	7.59	-12.21, 27.40	93%	<0.00001	REM	0.45
Total	5/5	307	154	153	8.66	-0.84, 18.17	89%	<0.00001	REM	0.07
10 min after SA										
MX *vs* BLK	2/2	140	70	70	16.16	10.51, 21.80	33%	0.22	FEM	<0.00001
MX *vs* Ephedrine	1/1	60	30	30	6.78	0.24, 13.32	NA	NA	NA	0.04
Total	3/3	200	100	100	13.31	6.53, 20.09	69%	0.04	REM	0.0001
30 min after SA										
MX *vs* BLK	1/1	60	30	30	7.00	-0.85, 14.85	NA	NA	NA	0.08
MX *vs* Ephedrine	1/1	67	34	33	11.65	6.87, 16.43	NA	NA	NA	<0.00001
Total	2/2	127	64	63	10.39	6.31, 14.47	0%	0.32	FEM	<0.00001
DBP										
Baseline										
MX *vs* BLK	2/2	140	70	70	-2.75	-6.74, 1.23	0%	0.83	FEM	0.18
MX *vs* Ephedrine	3/3	167	84	83	-1.45	-4.62, 1.71	0%	0.80	FEM	0.37
Total	5/5	307	154	153	-1.96	-4.44, 0.52	0%	0.95	FEM	0.15
5 min after SA										

MX *vs* BLK	2/2	140	70	70	6.37	-2.44, 15.19	84%	0.01	REM	0.16
MX *vs* Ephedrine	3/3	167	84	83	7.17	-2.41, 16.75	86%	0.0008	REM	0.007
Total	5/5	307	154	153	6.97	1.22, 12.73	81%	0.0003	REM	0.02
10 min after SA										
MX *vs* BLK	2/2	140	70	70	10.69	6.82, 14.57	55%	0.14	REM	<0.00001
MX *vs* Ephedrine	1/1	60	30	30	14.67	9.76, 19.58	NA	NA	NA	<0.00001
Total	3/3	200	100	100	11.82	8.29, 15.35	57%	0.10	REM	<0.00001
15 min after SA										
MX *vs* BLK	2/2	140	70	70	11.17	3.34, 19.00	94%	<0.0001	REM	0.005
MX *vs* Ephedrine	3/3	167	84	83	9.35	1.44, 17.26	79%	0.008	REM	0.02
Total	5/5	307	154	153	10.49	5.86, 15.12	85%	<0.0001	REM	<0.00001
30 min after SA										
MX *vs* BLK	1/1	60	30	30	8.00	3.13, 12.87	NA	NA	NA	0.001
MX *vs* Ephedrine	1/1	77	34	33	17.51	13.88, 21.14	NA	NA	NA	<0.00001
Total	2/2	127	64	63	14.11	11.21, 17.02	89%	0.002	REM	<0.00001
MAP										
Baseline										
MX *vs* BLK	1/2	80	40	40	-1.76	-4.09, 0.56	0%	0.87	FEM	0.14
MX *vs* Ephedrine	1/1	40	20	20	2.20	-7.35, 11.75	NA	NA	NA	0.65
Total	2/3	120	60	60	-1.54	-3.80, 0.72	0%	0.72	FEM	0.18
5 min after SA										
MX *vs* BLK	1/2	80	40	40	10.77	8.12, 13.43	0%	0.58	FEM	<0.00001
MX *vs* Ephedrine	1/1	40	20	20	-2.30	-12.08, 7.48	NA	NA	NA	0.64
Total	3/3	120	60	60	9.87	7.31, 12.44	70%	0.04	REM	<0.00001
15 min after SA										

MX *vs* BLK	1/1	36	17	19	9.83	6.18, 13.48	NA	NA	NA	<0.00001
MX *vs* Ephedrine	1/1	40	20	20	-2.30	-11.64, 7.04	NA	NA	NA	0.63
Total	2/2	76	37	39	8.22	4.82, 11.62	82%	0.02	REM	<0.00001
HR										
Baseline										
MX *vs* BLK	4/5	277	139	138	-1.06	-2.67, 0.55	19%	0.29	FEM	0.20
MX *vs* Ephedrine	3/3	167	84	83	1.47	-2.46, 5.40	0%	0.47	FEM	0.46
Total	7/8	444	223	221	-0.69	-2.18, 0.79	10%	0.35	FEM	0.36
5 min after SA										
MX *vs* BLK	3/4	220	110	110	-9.56	-14.56, -4.57	84%	0.0003	REM	0.0002
MX *vs* Ephedrine	3/3	167	84	83	-2.76	-10.00, 4.48	80%	0.007	REM	0.45
Total	6/7	387	194	193	-6.71	-11.65, -1.76	89%	<0.00001	REM	0.008
10 min after SA										
MX *vs* BLK	3/4	220	110	110	-6.97	-8.89, -5.05	33%	0.21	FEM	<0.00001
MX *vs* Ephedrine	1/1	60	30	30	-33.06	-39.26, -26.86	NA	NA	NA	<0.00001
Total	4/5	280	140	140	-9.25	-11.09, -7.42	94%	<0.00001	REM	<0.00001
15 min after SA										
MX *vs* BLK	4/4	233	116	117	-4.10	-5.31, -2.88	79%	0.003	REM	<0.00001
MX *vs* Ephedrine	3/3	160	80	80	-14.16	-17.62, -10.71	92%	<0.00001	REM	<0.00001
Total	7/7	393	196	197	-5.20	-6.34, -4.05	91%	<0.00001	REM	<0.00001
30 min after SA										
MX *vs* BLK	2/3	140	70	70	-5.78	-9.06, -2.49	36%	0.21	FEM	0.0006
MX *vs* Ephedrine	1/1	67	34	33	-17.05	-21.07, -13.03	NA	NA	NA	<0.00001
Total	3/4	207	104	103	-8.53	-14.75, -2.31	87%	<0.0001	REM	0.007

Abbreviations: DBP = diastolic blood pressure, FEM = fixed effect model, HR = heart rate, MAP = mean arterial pressure, NA = not applicable,

WMD = weighted mean difference, OR = odds ratio, REM = random effect model, SBP = systolic blood pressure, SA = spinal anesthesia, 95% CI = 95% confidence interval.
