# Supplementary material for: The efficacy and safety of pre-emptive methoxamine infusion in preventing hypotension by in elderly patients receiving spinal anesthesia: A PRISMA-compliant protocol for systematic review and meta-analysis
Source: Medicine (Baltimore). 2022 Dec 9;101(49):e32262. doi: 10.1097/MD.0000000000032262 (PMC9750677; doi:10.1097/MD.0000000000032262)
Supplement: Supplementary file 4 [file medi-101-e32262-s004.pdf]

Supplemental Table 4. Subgroup analysis of hemodynamic indexes in patients with intramuscular and intravenous injection

Outcomes	Trails/Comparisons(n)	Total(n)	MX(n)	CTL(n)	WMD	95%CI	I^2	Heterogeneity *P*	Model	Overall Effect p
SBP										
Baseline										
IV	5/5	306	153	151	-1.08	-5.21, 3.06	0%	0.95	FEM	0.61
IM	1/1	60	30	30	-2.00	-11.67, 7.67	NA	NA	NA	0.69
Total	6/6	364	183	181	-1.22	-5.02, 2.58	0%	0.98	FEM	0.53
5 min after SA										
IV	4/4	247	124	123	8.12	-3.87, 20.10	92%	<0.00001	REM	0.18
IM	1/1	60	30	30	11.00	3.66, 18.34	NA	NA	NA	0.003
Total	5/5	307	154	153	8.66	-0.84, 18.17	89%	<0.00001	REM	0.07
10 min after SA										
IV	2/2	140	70	70	10.68	3.63, 17.73	65%	0.09	REM	0.003
IM	1/1	60	30	30	20.00	11.90, 28.10	NA	NA	NA	<0.00001
Total	3/3	200	100	100	13.31	6.53, 20.09	69%	0.04	REM	0.0001
15 min after SA										
IV	5/5	304	153	151	11.58	0.26, 22.90	89%	<0.00001	REM	0.04
IM	1/1	60	30	30	14.00	5.62, 22.38	NA	NA	NA	0.001
Total	6/6	364	183	181	12.15	3.01, 21.30	86%	<0.00001	REM	0.009
30 min after SA										
IV	1/1	67	34	33	11.65	6.87, 16.43	NA	NA	NA	<0.00001
IM	1/1	60	30	30	7.00	-0.85, 14.85	NA	NA	NA	0.08
Total	2/2	127	64	63	10.39	6.31, 14.47	0%	0.32	FEM	<0.00001
DBP										
Baseline										

IV	4/4	247	124	123	-1.53	-4.74, 1.42	0%	0.93	FEM	0.31
IM	1/1	60	30	30	-3.00	-7.58, 1.58	NA	NA	NA	0.20
Total	5/5	307	154	153	-1.96	-4.44, 0.52	0%	0.95	FEM	0.15
5 min after SA										
IV	4/4	247	124	123	8.36	-1.74, 14.97	80%	0.002	REM	0.01
IM	1/1	60	30	30	2.00	-2.58, 6.58	NA	NA	NA	0.39
Total	5/5	307	154	153	6.97	1.22, 12.73	81%	0.0003	REM	0.02
10 min after SA										
IV	2/2	140	70	70	11.49	5.98, 17.01	73%	0.05	REM	<0.0001
IM	1/1	60	30	30	13.00	8.69, 17.31	NA	NA	NA	<0.00001
Total	3/3	200	100	100	11.82	8.29, 15.35	57%	0.10	REM	<0.00001
15 min after SA										
IV	4/4	247	124	123	11.84	7.26, 16.42	76%	0.0006	REM	<0.00001
IM	1/1	60	30	30	7.00	3.46, 10.54	NA	NA	NA	0.0001
Total	5/5	307	154	153	10.49	5.86, 15.12	85%	<0.0001	REM	<0.00001
20 min after SA										
IV	1/1	60	30	30	8.25	4.39, 12.11	NA	NA	NA	<0.0001
IM	1/1	60	30	30	10.00	4.91, 15.09	NA	NA	NA	<0.0001
Total	2/2	120	60	60	8.89	5.82, 11.96	0%	0.59	FEM	<0.00001
30 min after SA										
IV	1/1	60	30	30	8.00	3.13, 12.87	NA	NA	NA	0.001
IM	1/1	77	34	33	17.51	13.88, 21.14	NA	NA	NA	<0.00001
Total	2/2	127	64	63	14.11	11.21, 17.02	89%	0.002	REM	<0.00001
MAP										
15 min after SA										

IV	1/1	40	20	20	-2.30	-11.64, 7.04	NA	NA	NA	0.63
IM	1/1	36	17	19	9.83	6.18, 13.48	NA	NA	NA	<0.00001
Total	2/2	76	37	39	8.22	4.82, 11.62	82%	0.02	REM	<0.00001
HR										
Baseline										
IV	6/7	384	193	191	-1.06	-2.59, 0.48	0%	0.65	FEM	0.18
IM	1/1	60	30	30	5.00	-1.07, 11.07	NA	NA	NA	0.11
Total	7/8	444	223	221	-0.69	-2.18, 0.79	10%	0.35	FEM	0.36
5 min after SA										
IV	5/6	227	164	163	-7.16	-12.60, -1.71	89%	<0.00001	REM	0.010
IM	1/1	60	30	30	-4.00	-8.58, 0.58	NA	NA	NA	0.09
Total	6/7	387	194	193	-6.71	-11.65, -1.76	89%	<0.00001	REM	0.008
10 min after SA										
IV	3/4	220	110	110	-9.83	-11.78, -7.87	95%	<0.00001	REM	<0.00001
IM	1/1	60	30	30	-5.00	-10.32, 0.32	NA	NA	NA	0.07
Total	4/5	280	140	140	-9.25	-11.09, -7.42	94%	<0.00001	REM	<0.00001
15 min after SA										
IV	5/5	297	149	148	-5.81	-7.55, -4.07	94%	<0.00001	REM	<0.00001
IM	3/3	96	47	49	-4.73	-6.25, -3.22	77%	0.04	REM	<0.00001
Total	7/7	393	196	197	-5.20	-6.34, -4.05	91%	<0.00001	REM	<0.00001
20 min after SA										
IV	1/2	80	40	40	-8.23	-11.57, -4.90	0%	0.80	FEM	<0.00001
IM	1/1	60	30	30	3.00	-2.16, 8.16	NA	NA	NA	0.25
Total	2/3	140	70	70	-4.93	-7.73, -2.13	85%	0.002	REM	0.0006
30 min after SA										

IV	2/3	147	74	73	-10.61	-17.13, -4.08	86%	0.0007	REM	0.001
IM	1/1	60	30	30	-2.00	-7.06, 3.06	NA	NA	NA	0.44
Total	3/4	207	104	103	-8.53	-14.75, -2.31	87%	<0.0001	REM	0.007

Abbreviations: DBP = diastolic blood pressure, FEM = fixed effect model, HR = heart rate, IM = intramuscular injection, IV = intravenous injection, MAP = mean arterial pressure, NA = not applicable, WMD = weighted mean difference, OR = odds ratio, REM = random effect model, SBP = systolic blood pressure, SA = spinal anesthesia, 95% CI = 95% confidence interval.
