# Supplementary material for: The efficacy and safety of pre-emptive methoxamine infusion in preventing hypotension by in elderly patients receiving spinal anesthesia: A PRISMA-compliant protocol for systematic review and meta-analysis
Source: Medicine (Baltimore). 2022 Dec 9;101(49):e32262. doi: 10.1097/MD.0000000000032262 (PMC9750677; doi:10.1097/MD.0000000000032262)
Supplement: Supplementary file 5 [file medi-101-e32262-s005.pdf]

Supplemental Table 5. Effect of statistical model transformation on the combined effect value of outcome indicators

Outcomes	Heterogeneity		WMD/OR (95%)		Overall Effect p	
	I^2	p	FEM	REM	FEM	REM
5 min after SA SBP	89%	<0.00001	6.67 [3.79, 9.55]	8.66 [-0.84, 18.17]	<0.00001	0.07
15 min after SA MAP	53%	0.15	4.21 [1.02, 7.40]	2.72 [-4.01, 9.45]	0.010	0.43
20 min after SA HR	85%	0.002	-4.93 [-7.73, -2.13]	-4.58 [-11.72, 2.56]	0.0006	0.21

Abbreviations: FEM = fixed effect model, HR = heart rate, MAP = mean arterial pressure, WMD = weighted mean difference, OR = odds ratio, REM = random effect model, SBP = systolic blood pressure, SA = spinal anesthesia, 95% CI = 95% confidence interval.
