# Supplementary material for: The efficacy and safety of pre-emptive methoxamine infusion in preventing hypotension by in elderly patients receiving spinal anesthesia: A PRISMA-compliant protocol for systematic review and meta-analysis
Source: Medicine (Baltimore). 2022 Dec 9;101(49):e32262. doi: 10.1097/MD.0000000000032262 (PMC9750677; doi:10.1097/MD.0000000000032262)
Supplement: Supplementary file 6 [file medi-101-e32262-s006.pdf]

Supplemental Table 6. Sensitivity analysis (itemized elimination)

Outcomes	Excluded Trails	Heterogeneity		WMD/OR (95% CI)		Overall Effect p	
		I^2	*P*	FEM	REM	FEM	REM
10 min after SA SBP	[35]	84%	<0.00001	12.00 [6.91, 17.08]	13.17 [0.22, 26.11]	<0.00001	0.05
15 min after SA SBP	[33]	88%	<0.00001	16.07 [12.82, 19.32]	11.59 [0.25, 22.93]	<0.00001	0.05
5 min after SA DBP	[35]	84%	0.0003	6.11 [3.45, 8.78]	5.87 [-1.19, 12.93]	<0.00001	0.10
5 min after SA DBP	[48]	86%	0.0001	7.68 [4.82, 10.54]	7.24 [-0.63, 15.10]	<0.00001	0.07
5 min after SA MAP	[52]	82%	0.02	8.64 [5.29, 11.99]	-4.77 [-7.26, 16.80]	<0.00001	0.44
5 min after SA MAP	[52]	85%	0.010	9.63 [5.95, 13.31]	5.40 [-8.15, 18.94]	<0.00001	0.43
5 min after SA HR	[52]	91%	<0.00001	-10.56 [-11.96, -9.16]	-5.89 [-11.69, -0.09]	<0.00001	0.05
20 min after SA HR	[33]	0%	0.80	-8.23 [-11.57, -4.90]	-8.23 [-11.57, -4.90]	<0.00001	<0.00001
30 min after SA HR	[52]	91%	<0.00001	-9.67 [-12.18, -7.17]	-8.78 [-17.42, -0.13]	<0.00001	0.05

Abbreviations: DBP = diastolic blood pressure, FEM = fixed effect model, HR = heart rate, MAP = mean arterial pressure, NA = not applicable, WMD = weighted mean difference, OR = odds ratio, REM = random effect model, SBP = systolic blood pressure, SA = spinal anesthesia, 95% CI = 95% confidence interval.
