# Supplementary figures and images for: The efficacy and safety of pre-emptive methoxamine infusion in preventing hypotension by in elderly patients receiving spinal anesthesia: A PRISMA-compliant protocol for systematic review and meta-analysis
Source: Medicine (Baltimore). 2022 Dec 9;101(49):e32262. doi: 10.1097/MD.0000000000032262 (PMC9750677; doi:10.1097/MD.0000000000032262)

**Supplement Figure 1. Risk of bias graph**

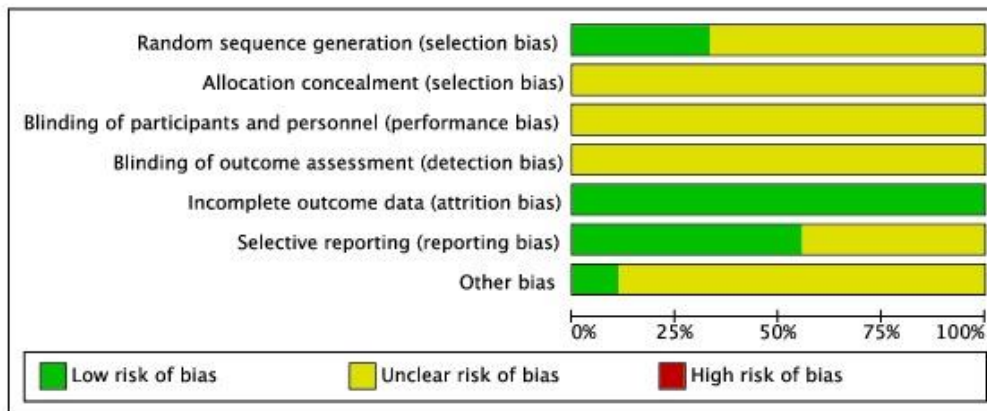

Supplement: Supplementary file 7 [file medi-101-e32262-s007.pdf]

**Supplement Figure 3.** Funnel plot for hypotension

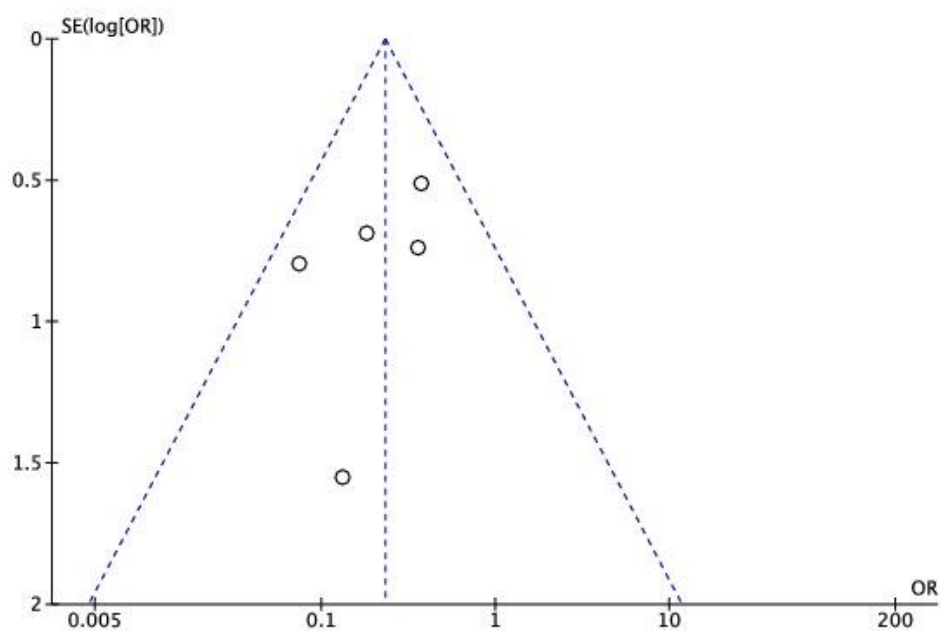

Supplement: Supplementary file 9 [file medi-101-e32262-s009.pdf]
